# Possible Emergence of Zika Virus of African Lineage in Brazil and the Risk for New Outbreaks

**DOI:** 10.3389/fcimb.2021.680025

**Published:** 2021-07-23

**Authors:** Sophia Martins Simon de Matos, André Ferreira Hennigen, Gabriela Elis Wachholz, Bruna Duarte Rengel, Lavinia Schuler-Faccini, Paulo Michel Roehe, Ana Paula Muterle Varela, Lucas Rosa Fraga

**Affiliations:** ^1^ Laboratory of Genomic Medicine, Experimental Research Center, Hospital de Clínicas de Porto Alegre, Porto Alegre, Brazil; ^2^ Laboratory of Virology, Department of Microbiology, Immunology and Parasitology, Institute of Health Sciences, Universidade Federal do Rio Grande do Sul, Porto Alegre, Brazil; ^3^ Postgraduate Program in Genetics and Molecular Biology, Department of Genetics, Universidade Federal do Rio Grande do Sul, Porto Alegre, Brazil; ^4^ Teratogen Information Service, Hospital de Clínicas de Porto Alegre, Porto Alegre, Brazil; ^5^ Postgraduate Program in Bioscience, Universidade Federal de Ciências da Saúde de Porto Alegre, Porto Alegre, Brazil; ^6^ Department of Morphological Sciences, Institute of Health Sciences, Universidade Federal do Rio Grande do Sul, Porto Alegre, Brazil; ^7^ Postgraduate Program in Medicine: Medical Sciences, Universidade Federal do Rio Grande do Sul, Porto Alegre, Brazil

**Keywords:** Congenital Zika Syndrome, epidemic, teratogenesis, animal models, viral emergence, birth defects

## Introduction

In 2015, an outbreak of Zika virus (ZIKV) infection caused a disproportionate increase in the number of cases of microcephaly in Brazil (de Oliveira et al., 2017). Although two major ZIKV lineages referred to as African lineage (ZIKV^AF^) and Asian lineage (ZIKV^AS^) have been identified, phylogenetic and genomic analyses pointed to those cases as caused by a ZIKV^AS^ (Faria et al., 2017). This link led scientists to not target ZIKV^AF^ on their investigations. Due to this, information about ZIKV^AF^ circulation in Brazil has been underestimated as well as its epidemic potential.

**Figure 1:**
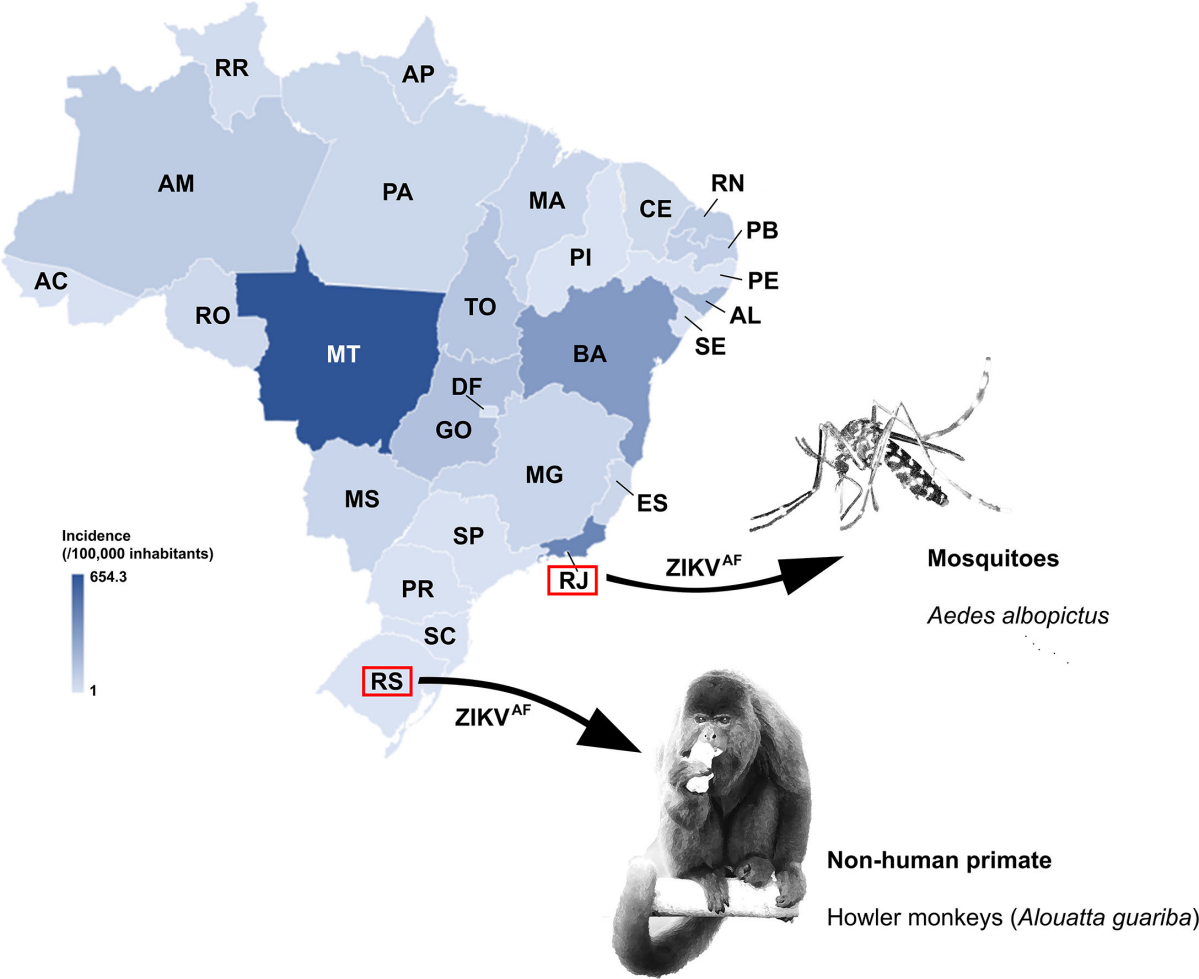
Incidence per 100,000 inhabitants of Zika cases in Brazil reported in 2016. Color gradient representing high (darker blue) to low ZIKV incidence (lighter blue). The Brazilian states where ZIKV^AF^ was identified in sylvatic area are also highlighted. Zika virus incidence data were recovered from Brazil Ministry of Health (2018). AC, Acre; AL, Alagoas; AP, Amapá; AM, Amazonas; BA, Bahia; CE, Ceará; DF, Distrito Federal; ES, Espírito Santo; GO, Goiás; MA, Maranhão; MT, Mato Grosso; MS, Mato Grosso do Sul; MG, Minas Gerais; PA, Pará; PB, Paraíba; PR, Paraná; PE, Pernambuco; PI, Piauí; RJ, Rio de Janeiro; RN, Rio Grande do Norte; RS, Rio Grande do Sul; RO, Rondônia; RR, Roraima; SC, Santa Catarina; SP, São Paulo; SE, Sergipe; TO, Tocantins.

Recently, a study published by Kasprzykowski et al. (2020) identified ZIKV^AF^ present among Brazilian ZIKV sequences. ZIKV^AF^ was identified in non-human primates (NHPs) and mosquitoes in the South and Southeast Brazilian regions, respectively (**Figure 1**) (de Almeida et al., 2019– Preprint publication; Alencar et al., 2021). At the same time, Aubry et al. (2021) showed ZIKV^AF^ being more transmissible in mosquitoes and more lethal in mice when compared to ZIKV^AS^. In addition, it was suggested that there was a high epidemic potential of ZIKV^AF^. Since South and Southeast Brazilian regions are geographically distant (> 1,500 km) and climatically different, and the ZIKV^AF^ was found from different sources, here we raise and bring concerns about the circulation of this lineage in Brazil and the risk it might represent as the cause of a new outbreak of disease in humans potentially associated with birth defects.

## Zika Virus: Epidemiological Profile and a Threat of New Outbreaks

The Zika virus epidemic spread quickly through South America, extending its range beyond the areas of recurrent transmission of arboviruses, including areas restricted by climatic barriers or low population density (Lowe et al., 2018). After the outbreak in 2015/2016, at least two smaller waves associated with ZIKV^AS^ were reported in 2017 and 2018 in two geographically distant and climatically different Brazilian regions, Southeast and Northern (Giovanetti et al., 2020; Iani et al., 2021). These sporadic outbreaks highlight the ongoing virus circulation, despite the considerable decrease in incidence of ZIKV-associated disease in human.

It is possible that the decline in prevalence of ZIKV disease is due to a buildup in herd immunity (Masmejan et al., 2020). Investigations performed in a city of Northeastern Brazil, a region highly affected by the virus, revealed that 63%–73% of the population had been exposed to the virus by the end of the 2015 outbreak (Netto et al., 2017). This provides substantial evidence to support the idea that the resulting herd immunity led ZIKV disease to become a rare event. Early epidemiological modeling investigations estimated that ZIKV infection rates during the pandemic in the Americas provided sufficient herd immunity to mitigate the risk of another large epidemic for at least another decade (Ferguson et al., 2016). However, a serological surveillance is essential to monitor ZIKV seroprevalence and to confirm this estimative. The persistency of neutralizing antibodies should be monitored through time to determine if there is or not a decline of immunity (Henderson et al., 2020). In addition, the degree of re-infection that humans might experience over time, in areas where infection is prevalent in mosquitoes, remains to be determined. The imbalance between the circulation of ZIKV in mosquitoes and the decline in herd immunity might dictate the risk for new outbreaks or for the reemergence of ZIKV. Herd immunity might not explain the decrease in ZIKV disease in humans in regions displaying low incidence. Other factors such as climatic condition, demographics, vector incidence, and mutation in the viral genome might also be influential (Masmejan et al., 2020; Liu et al., 2021).

In Brazil, ZIKV disease displayed uneven epidemiological outcomes across the regions **(** PAHO/WHO, 2017; Brazil Ministry of Health, 2018) (**Figure 1**), thus the epidemic potential of ZIKV to cause disease in humans and new outbreaks cannot be discarded. In 2016, the Northeast, Southeast, and Central-West regions were severely hit by the ZIKV epidemic accounting for 93.5% of the nationwide reported cases (16.11, 42.98 and 15.8%, respectively), whereas in Southern Brazil, only few cases of ZIKV infection (< 0.5%) had been reported (PAHO/WHO, 2017; Brazil Ministry of Health, 2018; Lowe et al., 2018) (**Figure 1**). Regions where most of the population has not had contact with the ZIKV may be especially vulnerable. Moreover, the official diagnostic tests for ZIKV infection have been performed by immunoassay (ELISA test) and/or by molecular assay (PCR), and none of these usual approaches allows for the determination of ZIKV lineages. Thus, all positive results are considered ZIKV^AS^, and it might contribute to ZIKV^AF^ being unnoticed by public health surveillance systems. Clearly, diagnostic methods should be revised to include strain differential tests, since the proportion of ZIKV^AF^ and ZIKV^AS^ circulating in Brazil is as yet unknown. Additionally, asymptomatic infections or absence of severe infection outcomes such as microcephaly or Congenital Zika Syndrome could have contributed to unnoticed ZIKV detection, regardless of the lineage.

In addition to uneven epidemiological outcomes of ZIKV disease, Brazil is a tropical country that displays favorable environmental and climatic conditions to sustain multiplication of mosquitoes as well as alternative reservoirs. Eventually, these vectors may be carrying ZIKV, regardless of the lineages and supporting a possible establishment of a sylvatic cycle, critical for the arbovirus maintenance (Lowe et al., 2018; Giovanetti et al., 2020). Recently, ZIKV^AF^ circulation in mosquitoes and NHPs in sylvatic areas in Brazil was evidenced (**Figure 1**) (de Almeida et al., 2019– Preprint publication; Alencar et al., 2021). This lineage displayed higher transmissibility in *Aedes aegypti* than ZIKV^AS^, and it was also verified to mosquitoes from South American (Aubry et al., 2021). Alencar et al. (2021) found *Ae. albopictus* naturally infected with ZIKV^AF^ in the Southeast of Brazil, recovered from the Atlantic Forest. The ZIKV sequence from *Ae. albopictus* was classified as ZIKV^AF^ by an automated sub-typing screening system developed by Kasprzykowski et al. (2020). This species is a recognized vector for arboviruses (Vazeille et al., 2010) and has been detected in all Brazilian urban and peri-urban areas (Carvalho et al., 2014), highlighting its high adaptability to different environmental conditions (Figueiredo, 2019). Unfortunately, in Brazil, the ongoing national entomological surveillance program reports on *Ae. aegypti* only, because this species is considered the main vector of transmission between humans and it is widely spread across Brazilian regions (Boyer et al., 2018). Moreover, the program targets exclusively the incidence of the vector and does not provide the virological investigation. Consequently, viruses circulating *Ae aegypti* as well as in other mosquito species that might act as potential vectors for arboviruses are not officially reported.

In free-living NHPs, ZIKV^AF^ was identified in *Alouatta guariba* in Southern Brazil (de Almeida et al., 2019 – Preprint publication). The viral genome was sequenced and it was observed to display 98% similarity with the MR766 strain, isolated in Uganda, a representative of the ZIKV^AF^. *Alouatta guariba* is a fairly common NHP in the Atlantic Forest (Culot et al., 2019). The species was hit hard in past yellow fever outbreaks as the genus is one of the most vulnerable to the disease (Moreno et al., 2015; dos Santos et al., 2020). The susceptibility of this species to ZIKV is yet to be described in detail, and predictive modeling systems estimated an alarming risk for the genus to be potentially involved as reservoirs in future reemergence of arboviruses events to humans (Han et al., 2019), not excluding ZIKV^AF^. In 2016, Brazilian research identified ZIKV^AS^ in marmosets and capuchin monkeys captured in Northeastern Brazil, a region largely affected by the virus (Favoretto et al., 2019). These peri-domestic animals were captured in proximity to humans in areas with reports of ZIKV-associated microcephaly cases during the epidemic period. Considering that ZIKV can circulate in NHPs, surveillance programs should include these potential reservoirs in wild environments, since the role of these animals in the epidemiology and prevalence of ZIKV remains unknown.

Since in Brazil there are temporally-consistent reports about the incidence of the vectors in all regions (Brazil Ministry of Health, 2018), and potential reservoirs, along with ZIKV^AF^ detection, even if only detected in the sylvatic area, the risk of a new outbreak caused by this lineage may not be discarded. Due to scarcity of data and testing negligence it is possible that the ZIKV^AF^ is also circulating in the country and could be spread, since its transmission efficiency in *Ae aegypti* is better than ZIKV^AS^.

## African Zika Virus Lineage: Experimental Findings

Experimental evidence *in vitro* and *in vivo* points towards higher transmissibility and pathogenicity of ZIKV^AF^ causing more productive and lethal infections than ZIKV^AS^ in cell culture (Anfasa et al., 2017; Gabriel et al., 2017; Sheridan et al., 2018; Smith et al., 2018) and more severe pathology in mice (Shao et al., 2017; Smith et al., 2018; Jaeger et al., 2019). In human cells, ZIKV^AF^ was able to infect and replicate in neural stem cells and astrocytes more efficiently than ZIKV^AS^ (Simonin et al., 2016). A clear disruption of normal gene expression levels was also observed in human prostate cells when infected by ZIKV^AF^ (Machado et al., 2021).

The ZIKV^AF^ has been reported for its high pathogenicity and teratogenic potential in animals model (Tripathi et al., 2017; Nunes et al., 2020). ZIKV^AF^ caused stronger infection, inflammation and transmission between fetuses than ZIKV^AS^ in a porcine model (Udenze et al., 2019). A comparison between the lineages also showed that ZIKV^AF^ induces higher embryo mortality in chicken embryos than ZIKV^AS^ (Willard et al., 2017). Moreover, Aubry et al. (2021) observed that ZIKV^AF^ was more pathogenic in immunocompromised adult mice, and in embryos of mice it was observed that intraplacental infection with ZIKV^AF^ caused subcutaneous edema, high levels of infection, and more severe phenotypes, leading to death. A study showed that vertical transmission of ZIKV^AF^ in mice leads to diminished fetal viability and viral RNA can be detected in different fetal tissues, including those related to brain/neurologic development (Vermillion et al., 2017).

Although Aubry et al. (2021) has shown ZIKV^AF^ having higher transmissibility and pathogenicity, so far, only ZIKV^AS^ has been related to disease and congenital anomalies in humans. Indeed, since ZIKV^AF^ has been reported as more virulent with the potential to cause serious and unrelated outcomes to life, including miscarriages and stillbirths, its effects in humans would be harder to be identified (Masmejan et al., 2020; Aubry et al., 2021; Lambrechts, 2021). However, taking into account ZIKV neurotropic ability and vertical transmission capacity (Aubry et al., 2021; Lambrechts, 2021), we cannot rule out that new outbreaks related to ZIKV^AF^ could increase the rate of malformations. Thus, such as apparently conflicting situation requires further investigation, mainly in areas where ZIKV^AF^ may be circulating.

## Conclusion

ZIKV has circulated for several decades with silent transmission causing sporadic outbreaks. So far, only ZIKV^AS^ has been associated with birth defects in humans. However, experimental data have also shown the teratogenic potential of ZIKV^AF^. Thus, in order to prevent new human outbreaks and potentially associated birth defects, close epidemiological vigilance and identification of ZIKV lineages circulating in mosquitoes, humans, and NPHs is of high importance. The detection of ZIKV^AF^ in mosquitoes and NHPs in a sylvatic environment in Brazil warns of the possibility of occurrence of a sylvatic cycle involving ZIKV^AF^. Mosquitoes and NHPs, which naturally inhabit the wild, can be easily introduced into urban and peri-urban regions, where half of the total Brazilian population resides. Additional studies and development of appropriate diagnostic assay capable of distinguishing viral lineages would be fundamental in establishing the role of such lineages in human and NHPs disease. Effective vector control programs should also be encouraged. These could aid in building a better understanding of the viral dynamics in urban and sylvatic environments, and prevent future ZIKV outbreaks. It is expected that such interventions may mitigate the impact of ZIKV on public health, avoiding disastrous outcomes, such as those of the 2016 ZIKV epidemic.

## Author Contributions

SMSM, AFH, GEW, and BDR performed the writing—draft. APMV and LRF led the manuscript and performed the writing—review. LS-F and PMR provided important comments. All authors contributed to the article and approved the submitted version.

## Funding

Study funded by Conselho Nacional de Desenvolvimento Científico e Tecnológico (CNPq)—Universal MCTIC/CNPq 2018 (project 424362/2018-0); and by the Fundo de Incentivo à Pesquisa e Eventos do Hospital de Clínicas de Porto Alegre (FIPE/HCPA) (Grant 2019-0649).

## Conflict of Interest

The authors declare that the research was conducted in the absence of any commercial or financial relationships that could be construed as a potential conflict of interest.

## Publisher’s Note

All claims expressed in this article are solely those of the authors and do not necessarily represent those of their affiliated organizations, or those of the publisher, the editors and the reviewers. Any product that may be evaluated in this article, or claim that may be made by its manufacturer, is not guaranteed or endorsed by the publisher.
